# The Use of Radioactive Marker as a Tool to Evaluate the Drug Release in Plasma and Particle Biodistribution of Block Copolymer Nanoparticles

**DOI:** 10.1155/2011/349206

**Published:** 2011-07-07

**Authors:** Sharon Johnstone, Steven Ansell, Sherwin Xie, Lawrence Mayer, Paul Tardi

**Affiliations:** Celator Pharmaceuticals Inc., 1779 W 75th Avenue, Vancouver, BC, Canada V6P 6P2

## Abstract

Diblock copolymer nanoparticles encapsulating a paclitaxel prodrug, Propac 7, have been used to demonstrate the usefulness of a nonmetabolizable radioactive marker, cholesteryl hexadecyl ether (CHE), to evaluate nanoparticle formulation variables. Since CHE did not exchange out of the nanoparticles, the rate of clearance of the CHE could be used as an indicator of nanoparticle stability in vivo. We simultaneously monitored prodrug circulation and carrier circulation in the plasma and the retention of CHE relative to the retention of prodrug in the plasma was used to distinguish prodrug release from nanoparticle plasma clearance. Nanoparticles labelled with CHE were also used to evaluate accumulation of nanoparticles in the tumour. This marker has provided relevant data which we have applied to optimise our nanoparticle formulations.

## 1. Introduction

Nanoparticle drug carriers improve the therapeutic benefit of chemotherapeutic agents by exploiting the enhanced permeability and retention (EPR) of solid tumours to improve localised chemotherapeutic drug accumulation ([1], as reviewed in [2]). One such drug carrier is the nanoparticle which forms through the self-association of amphiphilic diblock copolymers in aqueous environments. The hydrophobic core of these nanoparticles provides a reservoir which can serve as an in vivo carrier of hydrophobic drugs, and the hydrophilic shell confers compatibility with intravenous injection [3, 4]. Drug delivery effectiveness is dependent on circulation stability of the vehicle, the degree of retention of drug by the vehicle in the circulation, and on the level of accumulation of encapsulated drug in the targeted tumour [5]. Retention of encapsulated drug in the plasma during the early distribution phase of the carrier is crucial for maximising drug delivery to solid tumours since particles must remain in the circulation for at least 6 h to achieve the EPR effect [2]. Importantly, delivery is also tumour dependent given that the EPR effect is influenced by characteristics of the tumour such as the size and number of discontinuities in the endothelium, the interstitial tumour fluid pressure, and the tumour size (as reviewed in [6]).

Understanding the in vivo stability of the nanoparticle is vital to optimise nanoparticle formulation variables [1, 5]. Chemical characteristics of the stabiliser, size of the polymer, the size of nanoparticles, the drug/polymer ratio, and compatibility between the drug the nanoparticle core all have the potential to impact nanoparticle stability [7, 8]. For instance, since self-associations between the core-forming hydrophobic portion of the copolymer contributes to the stability of the nanoparticle, incorporating drugs into the core can impact these stabilising forces [9, 10]. Potentially, too high a drug/polymer ratio could increase the rate of release of drug from the nanoparticle in the circulation and thereby reduce amount of encapsulated drug reaching the tumour. Subtle changes in nanoparticle composition may then ultimately influence chemotherapeutic efficacy. There are many in vitro assays of nanoparticle stability; however these assays correlate poorly with in vivo nanoparticle circulation longevity and nanoparticle drug retention [7]. To understand the effect of our formulation variables on drug release in vivo and on the accumulation of the nanoparticle at the tumour site and on the release of entrapped agent at the tumour we needed to track the nanoparticle carrier and the entrapped agents simultaneously in vivo.

One indication of particle stability can be made by simply following the level of the entrapped drug circulating in the plasma over time. Since agents entrapped in vehicles are protected against metabolism all drug in the plasma can be considered to be drug retained in the carrier [11]. However loss of drug from the circulation does not distinguish between release of the drug from the carrier and drug cleared entrapped within the carrier. Historically, diblock copolymer nanoparticles have been followed by direct chemical labelling of the unit copolymer concomitant with demonstration of continued association of the labelled copolymer unit with the assembled nanoparticle in plasma samples [12, 13]. Following a single nanoparticle in this manner can be prohibitively labour intensive and thus tracing the plasma circulation and biodistribution of the carrier portion of the nanoparticle has rarely been done. We have elected to use an entrapped agent to follow our nanoparticles. Arguably an agent that is employed in such minute quantities as to not impact the stability of the core, that is very well retained by intact particles and that does not exchange into plasma lipids or proteins could prove useful to track nanoparticles in vivo. Cholesteryl hexadacyl ether (^3^[H] CHE) is a nonmetabolizable hydrophobic compound widely used to radioactively label liposomal nanoparticles and not known to exchange between membranes [14]. Here we have modified this approach by incorporating CHE into diblock copolymer nanoparticles and we demonstrate that monitoring the CHE provided credible and valuable information which aided in formulating diblock copolymer nanoparticles.

In a previous report, we described a series of paclitaxel prodrugs, Propac 1 to Propac 9, and detailed the in vitro stability, plasma circulation characteristics, and antitumour efficacy of nanoparticles formulations of these prodrugs [15]. Propac 7, is a prodrug in which paclitaxel is conjugated via a diglycolate linker to a C22 acyl chain. This prodrug is readily hydrolysed to paclitaxel in plasma. The nanoparticles were comprised of a stabiliser, 2.5K PEG-3K PS, a costabilising lipid (POPC), and a prodrug and the nanoparticles formed through flash coprecipitation. The lipid tail of the costabilising lipid inserts into the hydrophobic core of the nanoparticle while the polar headgroup localises the lipid to the interface between the hydrophobic core and the hydrophobic shell of the nanoparticle. The costabilising lipid is necessary to prevent hydrolytic cleavage of the prodrug during storage [15]. In order to optimise our carrier formulation we employed CHE to monitor nanoparticles prepared with a variety of diblock copolymers stabilisers and at a number of different component ratios. We then used this information to modify the nanoparticle drug retention characteristics and to investigate the impact of nanoparticle formulation on accumulation of the nanoparticles in tissues or in tumours. The simplicity of this approach has allowed us to generate a rapid and simple screening method which we used to assess the behaviour of many polymer formulations without necessitating the generation of specific covalently linked markers.

## 2. Materials and Methods 

### 2.1. Materials

The following copolymer stabilisers were obtained from Polymer Source (Dorval, Canada); polyethylene 2.5K glycol-polystyrene 3K (PS), polyethylene glycol 2K-poly (DL)-lactide 3.9K (PLA), and polyethylene glycol 5K-polycaprolactone 6K (PCL). The pegylated lipid 2-distearoyl-sn-glycero-3-phosphoethanolamine-N[amino(polyethylene glycol)-2000] (DSPE-PEG2K) was purchased from Avanti Polar Lipids (Alabaster Al, USA). Palmitoyl oleoyl phosphatidylcholine (POPC) was purchased from Northern Lipids, Burnaby, Canada. ^3^[H] cholesteryl hexadecyl ether, 45 Ci/mmol, (^3^[H] CHE) was obtained from Perkin Elmer Life and Analytical Sciences, Inc. (Waltham MA, USA). Ultima Gold scintillation fluid was purchased by PerkinElmer Life and Analytical Sciences (Boston, Massachusetts). Paclitaxel was purchased from Indena S.P.A., Milan, Italy. All solvents used were obtained from VWR International, Mississauga ON, Canada. Foxn1^nu^ athymic nude mice (7-8 weeks) were obtained from Harlan (Indianapolis IN, USA). HCT-116 tumour human colorectal carcinoma cells. paclitaxel-prodrug, 2′-O-(5′-O-docosanyldiglycoloyl)-paclitaxel (Propac 7) was synthesised as previously described [15]. 

### 2.2. Nanoparticle Formation and Characterization

Nanoparticle formation using flash coprecipitation has been described previously [15, 16]. Briefly, nanoparticles without drugs were prepared by dissolving 40 mg of polymer, PS, PLA, PCL, or DSPE-PEG2K, in 2 mL of ethanol : tetrahydrofuran (4 : 1 v/v). This solution was mixed with water using an impinging jet mixer with flow rates set at 6 mL/min (solvent) and 59 mL/min (water) using two Harvard Apparatus PHD 2000 syringe pumps. To prepare prodrug loaded nanoparticles Propac 7, POPC, and PS were combined at the indicated component ratio in ethanol: tetrahydrofuran (4 : 1 v/v) and mixed with water as for the drug-free nanoparticles. ^3^[H] CHE-labelled nanoparticles were prepared by adding CHE to the solvent mixture prior to nanoparticle formation. For plasma clearance studies, 2.5 *μ*Ci ^3^[H] CHE/40 mg of polymer was added (0.0000030 CHE/copolymer (w/w)). Nanoparticles for biodistribution studies were prepared with 4-fold higher amounts of radioactive label. Solvent was removed by dialysis in Spectrum Laboratories 3500 MW cut-off dialysis tubing (Spectrum Laboratories, Rancho Dominguez CA, USA) against water. Nanoparticles were concentrated in 300 mM sucrose by crossflow dialysis using a 100 kD (0.5 mm lumen, 60 cm path length) MidGee hoop cartridge (GE Healthcare Life Sciences, Piscataway NJ, USA). The final Propac 7 concentration was determined by HPLC. The prodrug encapsulation efficiency of this procedure was >90%. Nanoparticle size characteristics were determined by dynamic light scattering in water using a Malvern Zetasizer Nano-ZS (Malvern Instruments, Worcestershire, UK). Volume weighted hydrodynamic diameters were calculated using the Stokes-Einstein equation. 

### 2.3. Quantification of Paclitaxel Prodrug

Propac 7 was quantified on a Waters HPLC using a Phenomenex SynergiFusion analytical column. Plasma samples or nanoparticle suspensions (50 *μ*L) were mixed with 150 *μ*L of diluents (methanol : acetonitrile, 2 : 1 v/v) by vigorous vortexing and Propac 7 was recovered in the 10000 × g supernatant. The mobile phase consisted of a linear gradient from methanol/10 mM sodium acetate buffer (pH 5.6) (70 : 30 v/v) to 100% methanol at a flow rate of 1 mL/min. Column temperature was set at 30°C. A 20 *μ*L sample was injected onto the column and peak area was monitored by UV detection at 227 nm. Samples in the autosampler were maintained at 4°C prior to HPLC analysis.

### 2.4. Column Chromatography of Nanoparticles

Separation of nanoparticles from the bulk protein fraction was performed on a 20 cm × 1 cm Sepharose 4B (GE Healthcare Life Sciences, Piscataway NJ, USA) column equilibrated within HEPES buffered saline, pH 7.4 at a flow rate of 0.5 mL/min. Plasma samples (200 *μ*L) were loaded onto the column and 150 *μ*L fractions were collected and aliquots of each fraction were analyzed for ^3^[H] CHE in Ultimagold scintillation fluid (Perkin Elmer, Norwalk CT, USA) or counted on a Beckman-Coulter LS 6500 scintillation counter and for protein using Pierce microBCA assay (Thermoscientific Rockford IL, USA) and read using a Victor Multilabel plate reader (Perkin Elmer, Norwalk CT).

### 2.5. Pharmacokinetic Profile

All animal experiments were conducted according to protocols approved by the University of British Columbia's Animal Care Committee and in accordance with the current guidelines established by the Canadian Council of Animal Care. Nanoparticles were sterile filtered through 0.22 micron filters then injected into mice via the tail vein at a dose of 10 *μ*L/g body weight up to a maximum of 250 *μ*L. Whole blood was collected into EDTA coated microtainer tubes (BD Biosciences Bedford, MA, USA). Plasma was collected by centrifugation of the tubes at 2800 rpm for 10 minutes and stored −20°C until analysis. The quantity of Propac 7 in (50 *μ*L) aliquots was analyzed by HPLC. A second 50 *μ*L aliquot of each sample was measured for nanoparticle carrier by monitoring the ^3^[H] CHE by liquid scintillation counting with a LS 6500 Multi-Purpose Scintillation Counter; Beckman Coulter Canada Inc. (Mississauga ON, Canada). 

We determined the plasma half-life, the time to reach 1/2 of the initial Propac or 1/2 of the initial nanoparticle concentration, from the plasma concentration versus time curve. The initial nanoparticle concentration was calculated using the plasma volume. We have previously determined the plasma volume of Foxn1^nu^ mice averages 0.043 mL per gram of body weight up to a body weight of up to 25 g. 

### 2.6. Tumour and Tissue Accumulation of Radioactive Nanoparticles

Athymic nude Foxn1^nu^ Mice were injected s.c. with 2 × 10^6^ colorectal carcinoma HCT-116 cells. Tumour volumes were determined using digital callipers using the equation (length × width^2^)/2. ^3^[H] CHE-labelled nanoparticles, Propac 7/POPC/PS 1 : 1 : 2 (w/w/w) were injected when tumours reached an average size of 200 mm^3^. Tumours, tissue, and blood were collected at 1, 4, 8, 16, 24, and 48 hours post injection. Tissues and tumours were washed in ice cold saline and immediately frozen. Blood was centrifuged and the plasma fraction was recovered and stored frozen at −20°C until analysis. Tissue samples were digested in solvable (GE Health) at 60°C overnight then decolourised with H_2_0_2_. ^3^[H] CHE levels were determined by liquid scintillation counting.

### 2.7. Statistical Analysis

Data values are reported as mean ± standard deviation (SD). A standard one-way analysis of variance (ANOVA) was used to determine statistically significant differences from the mean. *P* < 0.05 was considered significant for all statistical tests.

## 3. Results and Discussion

### 3.1. Nanoparticle Formation and Characterisation

We have previously described paclitaxel prodrug nanoparticles generated through flash coprecipitation [15]. When the initial total concentration of the prodrug, colipid, and diblock copolymer stabiliser in the solvent was less than 40 mg/mL, this procedure produced small, homogeneous nanoparticles with a prodrug trapping efficiency of >90%. All nanoparticle solutions were clear with no precipitate and no visible haze. Nanoparticle size characteristics were determined by dynamic light scattering in water. Nanoparticles prepared without prodrug were 10–20 nm in diameter by DLS. Propac 7/POPC/PS 1 : 1 : 2 (w/w/w) nanoparticles had a mean diameter of 20–30 nm and unimodal size distributions suggesting a single population of nanoparticles. The prodrug stability was monitored by HPLC and the particle physical stability over time was monitored by DLS. When maintained at 4°C for 11 weeks, less than 5% of Propac 7 was hydrolysed and nanoparticle size increased by less than 15% [15].

### 3.2. CHE as a Nanoparticle Marker In Vivo

**Figure 1:**
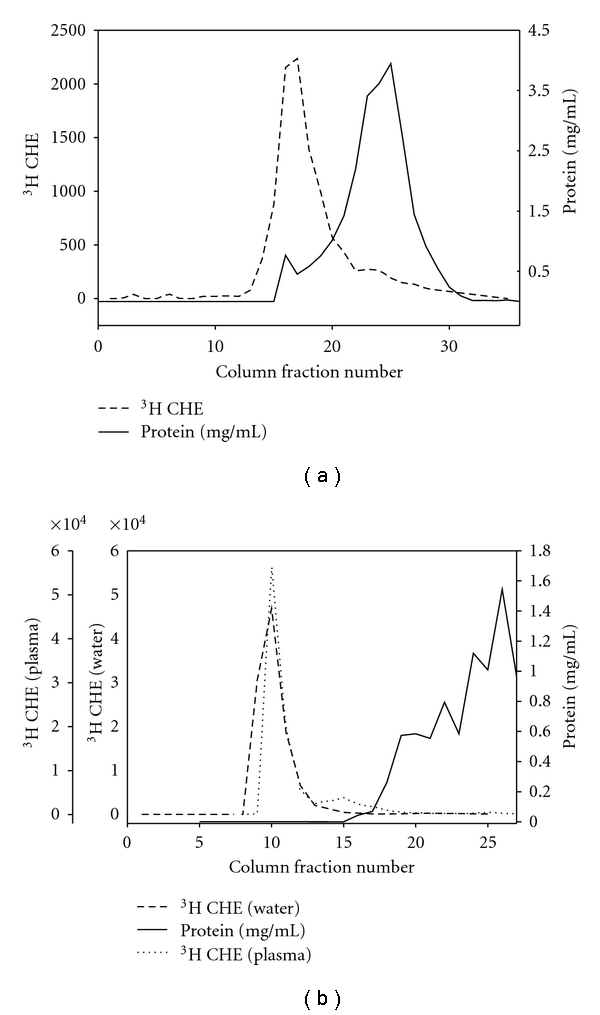
Column chromatography of nanoparticles. (a) Plasma from mice injected i.v. with ^3^[H] CHE-labelled Propac 7/POPC/PS 1 : 1 : 2 nanoparticles fractionated on a sepharose 4B column in HEPES buffered saline, pH 7.4. Column aliquots of were assayed for protein and ^3^[H]. The concentration of protein and ^3^[H] CHE for each fraction are plotted. (b) Plasma from mice injected i.v. with ^3^[H] CHE-labelled DSPE-2K nanoparticles were incubated either in water at room temperature or in mouse plasma at room temperature for one hour then fractionated on a sepharose 4B column. Column aliquots of were assayed for protein and ^3^[H]. The concentration of protein and ^3^[H] CHE for each fraction are plotted.

We used column chromatography to demonstrate the strong retention of CHE in nanoparticles during both in vitro and in vivo exposures to plasma and we provide examples of each in Figure 1. 

We prepared a 20 cm sepharose 4B column and established that nanoparticles eluted before the bulk protein fraction on this column. Nanoparticles incubated with mouse plasma at 37°C for one hour in vitro gave the same ^3^[H] elution profile as control nanoparticles (in water) (data not shown).  Figure 1(a) shows data from an in vivo experiment in which ^3^[H] CHE–labelled Propac 7/POPC/PS 1 : 1 : 2 (w/w/w) nanoparticles were injected i.v. into mice and plasma was recovered 24 hours later. Plasma was immediately chromatographed on the column and aliquots of the collected fractions were assayed for protein or ^3^[H]. The ^3^[H] chromatographic elution profile of nanoparticles recovered from the plasma were unchanged from that of the control nanoparticles. These data suggest that ^3^[H] CHE remained associated with our nanoparticles in plasma and did not exchange out of the nanoparticles and associate with plasma lipoproteins in vivo or in vitro. 

We strengthened the contention that CHE does not transfer to plasma proteins or lipids by incubating relatively unstable DSPE-PEG2K nanoparticles with plasma in vitro. DSPE-PEG2K nanoparticles have transition temperature of 15°C, and thus the lipid chains assume a disorder state well below physiological temperatures [17]. In the presence of bovine serum albumin protein or human plasma, DSPE-PEG2K nanoparticles disaggregation to monomers begins within minutes [18]. DSPE-PEG2K nanoparticles were chromatographed on a sepharose 4B column and the elution profile of the radioactivity is shown in Figure 1(b). The ^3^[H] associated with DSPE-PEG2K nanoparticles elutes as a single sharp peak. We then incubated DSPE-PEG2K nanoparticles in mouse plasma for one hour at room temperature and chromatographed this sample on the same sepharose 4B column. Aliquots of these collected fractions were assayed for protein or ^3^[H] and the elution profile is also shown in Figure 1(b). The bulk of the ^3^[H] coelutes with the nanoparticles; however a trailing shoulder peak appears in the serum treated chromatograph is indicative of some degree of nanoparticle disruption. Importantly radioactivity is not found associated with the bulk protein fraction implying that the CHE did not redistribute to the lipoproteins.

### 3.3. ^3^H-CHE Monitoring Plasma Clearance of Various Stabilisers

**Figure 2:**
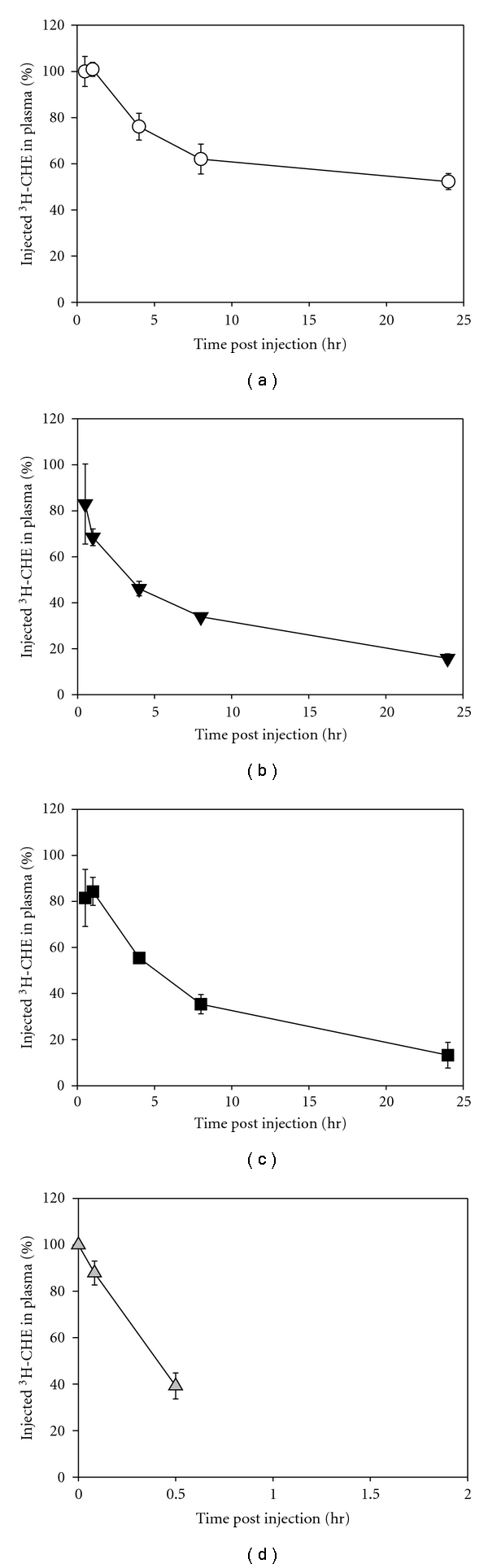
Plasma clearance of alternate polymer stabilisers. (a) ^3^[H] CHE-labelled PS. (b) ^3^[H] CHE-labelled PLA. (c) ^3^[H] CHE-labelled PCL. (d) ^3^[H] CHE-labelled DSPE-PEG2K. Each data point represents the mean (± standard deviation) of three mice.

We have found ^3^[H] CHE generally applicable to correlating nanoparticle formulation variables and in vivo plasma circulation including varying the stabilisers. We prepared ^3^[H] CHE-labelled nanoparticles with various stabilisers, in this instance, without colipid or prodrug (Table 1) and the plasma circulation characteristics of these nanoparticles are shown in Figure 2. The data from PS nanoparticles is shown in Figure 2(a). Approximately 50% of the ^3^[H] label remained 24 hours after injection, suggesting very stable, long circulating nanoparticles are formed with this copolymer. The more rapid clearance of  ^3^[H] associated with PLA and PCL (Figures 2(b) and 2(c), resp.) suggests that these nanoparticles have lower in vivo stability than the PS nanoparticles. Finally, we included DSPE-PEG2K nanoparticles, which have been demonstrated to be unstable in plasma in vitro [17, 18]. We contend that the in vivo stability will also be poor for these nanoparticles and should clear very rapidly from the circulation. We also predicted that if CHE from DSPE-PEG2K nanoparticles exchanged into plasma, we would observe a contradictorily prolonged ^3^[H] circulation. The rapid in vivo clearance of  ^3^[H] associated with the DSPE-PEG2K nanoparticles Figure 2(d) suggested that ^3^[H] CHE lost from these nanoparticles did not exchange to plasma lipids or lipoproteins.

### 3.4. Simultaneous Monitoring of Circulating Nanoparticle Formulation and Prodrug Release

**Figure 3:**
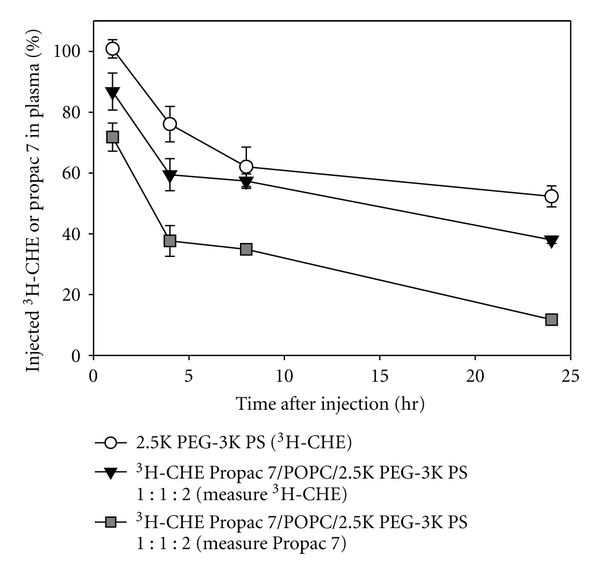
Plasma ^3^[H] clearance of  ^3^[H] CHE-labelled PS nanoparticles or plasma clearance of CHE-labelled Propac 7/POPC/PS 1 : 1 : 2 nanoparticles. The ^3^[H] CHE plasma profile is shown for the PEG-3K PS nanoparticles and both the ^3^[H] CHE-labelled and Propac 7 profiles are shown for the Propac 7/POPCPS nanoparticles. Each data point represents the mean (± standard deviation) of three mice. Clearance of Propac 7/POPC/2.5K PEG-3K PS is significantly faster than drug-free nanoparticles (*P* < 0.05 at each time points).

To develop an assay to monitor the effect of formulation parameters on prodrug release in vivo, we needed to monitor nanoparticle and prodrug plasma circulation simultaneously and independently. We prepared ^3^[H] CHE-labelled PS nanoparticles with prodrug and colipid; Propac 7/POPC/PS 1 : 1 : 2 (w/w/w). These nanoparticles were injected i.v. into mice and plasma was assayed for both ^3^[H] and Propac 7. The circulation profiles of the ^3^[H] and the prodrug are shown in Figure 3. To aid with the interpretation of data, we have reproduced the data for the nanoparticles without colipid or prodrug (Figure 2(a)). These results provided information about the nanoparticle stability and about prodrug retention in vivo. First, by comparing the circulation of  ^3^[H] associated with nanoparticles with and without prodrug, we were able to gauge the effect of encapsulating agents into the core of the nanoparticle. Long ^3^[H] circulation times were observed when ^3^[H] CHE was incorporated into nanoparticles without prodrug (*t*_1/2_ > 24 hours). A significant shortening of  ^3^[H] circulation time was observed when colipid and Propac 7 were introduced into the nanoparticles (*t*_1/2_ 14 hours). We attributed the enhanced clearance of ^3^[H] associated with prodrug loaded nanoparticles to a less stable nanoparticle formed when the prodrug was incorporated into the nanoparticle. Secondly, by simultaneously monitoring ^3^[H] and Propac 7 in prodrug loaded nanoparticles we were able to demonstrated prodrug clearance was faster than particle clearance. This suggested that the prodrug clearance occurred through both release of the prodrug from the nanoparticle and nanoparticle clearance. It is noteworthy that we have not always observed prodrug in plasma clearing faster than ^3^[H]. In a previous report we observed prodrug cleared at a rate equal to that of the ^3^[H] [15]. This occurred when nanoparticles were loaded with a nonmetabolisable paclitaxel drug conjugate, suggesting that in this instance all prodrug was being cleared still associated with the nanoparticle. 

**Figure 4:**
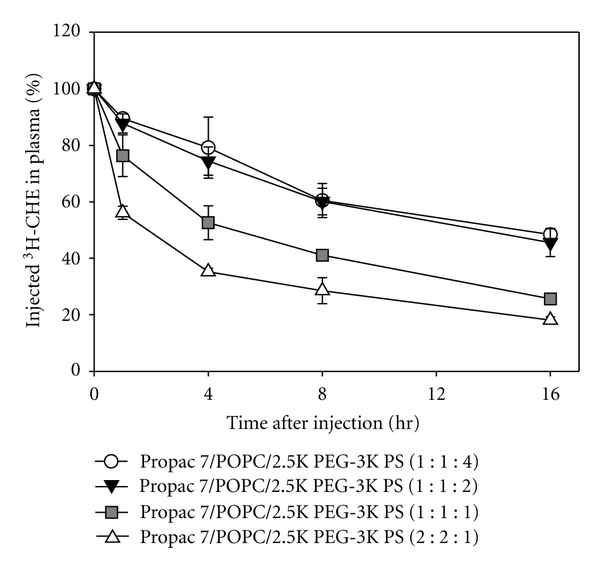
Plasma ^3^[H] profile of  ^3^[H] CHE-labelled Propac 7/POPC/PS prepared at different polymer ratios. ^3^[H] CHE-labelled nanoparticles were prepared with 4 different ratios of Propac 7 and PS. Data for ^3^[H] levels is plotted. Each data point represents the mean (± standard deviation) of three mice. Clearance of Propac 7/POPC/PS 1 : 1 : 1 and 2 : 2 : 1 nanoparticles is significantly faster than 4 : 1 : 1 nanoparticles (*P* < 0.05 at each time points).

In a further set of experiments we demonstrated that we could monitor the effect of formulation composition on particle clearance. We prepared a series of  ^3^[H] CHE-labelled Propac 7/POPC/PS nanoparticles with component weight ratios of 1 : 1 : 4, 1 : 1 : 2, 1 : 1 : 1, and 2 : 2 : 1 (w/w/w) (Table 2). The mean diameters of the nanoparticles increased as the prodrug/polymer ratio increased. The data for ^3^[H] plasma clearance following injection of these nanoparticles is shown in Figure 4. The particles with the lowest amount of prodrug relative to polymer, Propac 7/POPC/PS 1 : 1 : 4 (w/w/w) had the smallest particle sizes and long ^3^[H] CHE-circulation lifetimes. Increasing the prodrug/polymer ratio in Propac 7/POPC/PS 1 : 1 : 2 (w/w/w) generated nanoparticles with 30% larger diameters, however the plasma clearance of the ^3^[H] was unchanged relative to the Propac 7/POPC/PS 1 : 1 : 4 (w/w/w) nanoparticles. This suggests that in vivo stability of these particles was not impacted by the increase in prodrug. Nanoparticles with Propac 7/POPC/PS 1 : 1 : 1 and 2 : 2 : 1 (w/w/w) formed larger nanoparticles and had faster ^3^[H] CHE-plasma clearances. These results suggested that the nanoparticles formed with this diblock copolymer stabiliser have a tolerance limit for the amount of prodrug which can be incorporated into the hydrophobic core. This information was used to determine the maximum nanoparticle drug content obtainable with minimal impact of nanoparticle stability.

In addition to varying component ratios and stabilisers, we have used ^3^[H] CHE labelling to evaluate the role of alternate costabilisers (vitamin E-succinate or phosphatidylglycerol) [15], and for nanoparticles loaded with different paclitaxel prodrugs or with hydrophobic prodrugs of other chemotherapeutic agents (gemcitabine and doxorubicin) (not shown). The loading capacity of nanoparticles is likely unique for each diblock copolymer and different for every drug. The ability to trace the nanoparticle in vivo is a valuable tool for choosing stabilisers and determining optimal loading ratios. Importantly, labelling nanoparticles by physically incorporating CHE is technically quite simple.

### 3.5. Tissue and Tumour Accumulation of Nanoparticles

**Figure 5:**
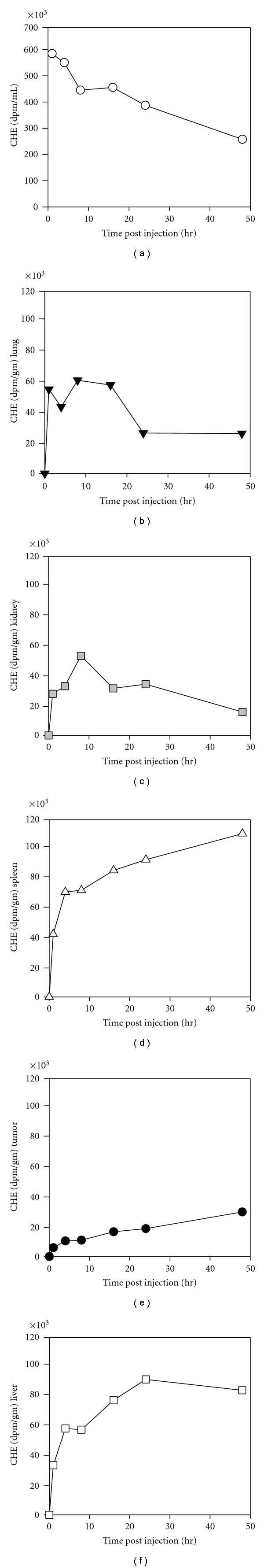
Plasma clearance and tissue accumulation of  ^3^[H] CHE-labelled Propac 7/POPC/PS nanoparticles. Plasma ^3^[H] profile and ^3^[H] tissue accumulation from HCT116 tumor-bearing mice injected with ^3^[H] CHE-labelled Propac 7/POPC/PS 1 : 1 : 2 (6 mg/kg Propac 7). Only ^3^[H] was measured. Data points are the average of two mice. (a) plasma, (b) lung, (c) kidney, (d) spleen, (e) tumor, and (f) liver.

To further evaluate the utility of this nanoparticle labelling technique we monitored plasma clearance and tumour accumulation of  ^3^[H] CHE-labelled nanoparticles. The goal was to try to understand the effect of formulation variables and dosing schedule on efficacy. To demonstrate that we could monitor nanoparticle accumulation in tissues and tumours, ^3^[H] CHE-labelled Propac 7/POPC/PS nanoparticles 1 : 1 : 2 (w/w/w) were injected into HCT-116 tumour-bearing mice. The plasma and tissues were harvested and the ^3^[H] levels were measured at various times. The results in Figure 5 shows plasma clearance of ^3^[H] associated with prodrug loaded nanoparticles (Figure 5(a)) and accumulation of  ^3^[H] in the organs and the tumours of the same mice over a 48-hour period (Figures 5(b)–5(f)). Notably, ^3^[H] accumulated in HCT-116 tumours for more than 48 hours underscoring the need for stable nanoparticles to maximise chemotherapeutic efficacy.

We have employed ^3^[H] CHE-labelled nanoparticles in order to compare accumulations in large and small tumours of same cell origin and to assess nanoparticle accumulation in different types of tumour (not shown). The reliance of nanoparticle tumour accumulation on the EPR effect makes tumour accumulation subject to variables such as the degree of tumour vascularization. The accumulation of CHE-labelled nanoparticles was useful to optimise dosing schedule and to understand the impact of nanoparticle composition on tumour efficacy. 

## 4. Conclusion

The in vivo stability of controlled release formulations critically impacts biodistribution and the pharmacokinetics of the encapsulated agent. It is imperative to characterise the effect of formulation variables on carrier stability and drug retention. Unfortunately in vitro assays of nanoparticle stability do not predict the stability of nanoparticles in vivo and labelling of diblock copolymers can be quite labour intensive. We have used entrapped radioactive agents to label our nanoparticles for in vivo assessment of nanoparticle stability. We have found that this label provided relevant data which accelerated screening of many variables and which aided in nanoparticle component selection and optimising component ratios. Furthermore, we further test selected formulations in efficacy experiments to substantiate our formulation choices made using plasma clearance data.

As a caution, one must confirm that a noncovalently incorporated nanoparticle label remains associated with the nanoparticle in plasma. We verified this by running size exclusion chromatographic columns which separated our nanoparticles from the bulk protein of the plasma and in every instance, all the ^3^[H] co-eluted from the column with nanoparticles. We have never observed transfer of ^3^[H] to the plasma fraction. One other potential flaw is an underestimation of nanoparticle stability if the ^3^[H] CHE is rapidly released and clearance of the nanoparticles themselves remain intact. This is a limitation that cannot be solved without directly labelling the copolymer. The most meaningful data will ultimately be the antitumour efficacy.

## Figures and Tables

**Table 1:** Stabilisers ^3^[H] CHE-labelled nanoparticles.

Polymers	Size nm (diameter)
PEG2.5-PS3K	16.3
PLA3.9K-PEG2K	9.3
PCL6K-PEG5K	18.9
DSPE2K-PEG	10

**Table 2:** Component weight ratios of formulations of Propac 7. Diameter of particles is the volume weighted average diameter and Zave of the diameter.

Formulation	w/w/w	Diameter nm	Zave nm
Propac 7/POPC/PS	1 : 1 : 4	20.9	23.8
Propac 7/POPC/PS	1 : 1 : 2	26.6	31.2
Propac 7/POPC/PS	1 : 1 : 1	34.9	40.5
Propac 7/POPC/PS	2 : 2 : 1	43.6	54.8

## Abbreviations

CHE: Cholesteryl hexadecyl ether
PEG: Polyethylene glycol
PLA: Poly (DL)-lactide
PLC: Polycaprolactone
DSPE: 2-distearoyl-sn-glycero-3-phosphoethanolamine
POPC: Palmitoyl oleoyl phosphatidylcholine
PS: Polystyrene.
